# Activity of the multikinase inhibitor dasatinib against ovarian cancer cells

**DOI:** 10.1038/sj.bjc.6605381

**Published:** 2009-10-27

**Authors:** G E Konecny, R Glas, J Dering, K Manivong, J Qi, R S Finn, G R Yang, K-L Hong, C Ginther, B Winterhoff, G Gao, J Brugge, D J Slamon

**Affiliations:** 1 Division of Hematology-Oncology, Department of Medicine, David Geffen School of Medicine, University of California Los Angeles, Los Angeles, CA, USA; 2 Division of Gynecologic Surgery, Department of Obstetrics and Gynecology, Mayo Clinic, Rochester, MN, USA; 3 Department of Cell Biology, Harvard Medical School, Boston, MA, USA

**Keywords:** Src, Eph2A, FAK, uPA, dasatinib, ovarian cancer

## Abstract

**Background:**

Here, we explore the therapeutic potential of dasatinib, a small-molecule inhibitor that targets multiple cytosolic and membrane-bound tyrosine kinases, including members of the Src kinase family, EphA2, and focal adhesion kinase for the treatment of ovarian cancer.

**Methods:**

We examined the effects of dasatinib on proliferation, invasion, apoptosis, cell-cycle arrest, and kinase activity using a panel of 34 established human ovarian cancer cell lines. Molecular markers for response prediction were studied using gene expression profiling. Multiple drug effect/combination index (CI) isobologram analysis was used to study the interactions with chemotherapeutic drugs.

**Results:**

Concentration-dependent anti-proliferative effects of dasatinib were seen in all ovarian cancer cell lines tested, but varied significantly between individual cell lines with up to a 3 log-fold difference in the IC_50_ values (IC_50_ range: 0.001–11.3 *μ*mol l^−1^). Dasatinib significantly inhibited invasion, and induced cell apoptosis, but less cell-cycle arrest. At a wide range of clinically achievable drug concentrations, additive and synergistic interactions were observed for dasatinib plus carboplatin (mean CI values, range: 0.73–1.11) or paclitaxel (mean CI values, range: 0.76–1.05). In this study, 24 out of 34 (71%) representative ovarian cancer cell lines were highly sensitive to dasatinib, compared with only 8 out of 39 (21%) representative breast cancer cell lines previously reported. Cell lines with high expression of Yes, Lyn, Eph2A, caveolin-1 and 2, moesin, annexin-1, and uPA were particularly sensitive to dasatinib.

**Conclusions:**

These data provide a clear biological rationale to test dasatinib as a single agent or in combination with chemotherapy in patients with ovarian cancer.

Ovarian cancer is a leading cause of cancer death among women in the United States and Western Europe and has the highest mortality rate of all gynaecologic cancers. Despite the fact that maximal cytoreductive surgery followed by combination chemotherapy increases 5-year survival rates, the overall survival gains have been modest with low overall cure rates (Aletti *et al*, 2007). Thus, novel systemic treatment approaches are urgently needed. Src is the prototypic member of a family of nine non-receptor tyrosine kinases (Src, Lyn, Fyn, Lck, Hck, Fgr, Blk, Yrk, and Yes) that has a key role in many cellular signalling pathways (reviewed in Thomas and Brugge, 1997). Src family kinase (SFK) proteins regulate four main cellular functions that ultimately control the behaviour of transformed cells: cell proliferation, adhesion, invasion, and motility (Summy and Gallick, 2003; Yeatman, 2004). Interactions with activated EGFR (Tice *et al*, 1999), HER2 (Muthuswamy *et al*, 1994), fibroblast growth factor receptor (Landgren *et al*, 1995), or hepatocyte growth factor (Mao *et al*, 1997) can result in Src activation, most likely by disrupting the intramolecular interactions that hold Src in a closed and inactive configuration (Thomas and Brugge, 1997). Importantly, aberrant expression and activation of the SFKs have been described in human ovarian cancer cell lines (Budde *et al*, 1994) and clinical samples (Wiener *et al*, 2003). A recent study used gene expression signatures that define the status of Src signalling pathways to predict the probability of Src pathway activation in 119 patients with ovarian cancer (Dressman *et al*, 2007). Approximately half of the tumours examined in this study showed Src pathway deregulation. Furthermore, those patients with a deregulated Src pathway also showed the worst prognosis.

Dasatinib is a small molecule that inhibits not only recombinant kinase domains of SFKs such as Src, Lck, and Yes, but also other tyrosine kinases such as Abl, c-Kit, and platelet-derived growth factor receptor-*β* (PDGFR-*β*) by 50% (IC_50_) at low nanomolar concentrations (Lombardo *et al*, 2004). Moreover, recent studies have also identified focal adhesion kinase (FAK; Bantscheff *et al*, 2007) and EphA2 (Huang *et al*, 2007) as direct targets of dasatinib. Focal adhesion kinase is a non-receptor tyrosine kinase involved in the regulation of cell adhesion, survival, and migration, which itself can be activated by Src (Fincham and Frame, 1998; Schaller, 2001). Importantly, preclinical studies indicate that FAK has a significant role in ovarian cancer cell migration and invasion (Sood *et al*, 2004). The Eph receptor tyrosine kinases and ephrin ligands have been studied extensively for their roles in developmental processes. However, in recent years, Eph receptors and ephrins have been found to be integral players in cancer formation and progression (Wykosky and Debinski, 2008). Eph2A, as well as other members of the Eph receptor tyrosine kinase family, have been associated with advanced ovarian cancer and poor clinical outcome (Thaker *et al*, 2004; Wu *et al*, 2006; Kumar *et al*, 2007; Alam *et al*, 2008). On the basis of these data, we hypothesise that dasatinib, which targets SFKs as well as FAK and Eph2A, may show significant preclinical activity in ovarian cancer cells supporting its further clinical evaluation. Thus far, dasatinib has proven to be clinically active in patients with imatinib-refractory chronic myelogenous leukaemia and Philadelphia chromosome-positive ALL (Talpaz *et al*, 2006). Moreover, dasatinib has also recently shown preclinical activity in prostate cancer cells (Nam *et al*, 2005), basal-like breast cancer cells (Finn *et al*, 2007), melanoma cells (Eustace *et al*, 2008), colon cancer cells (Serrels *et al*, 2006), and head and neck squamous cancer cells or non-small-cell lung cancer cells (Johnson *et al*, 2005; Song *et al*, 2006). Inhibitory effects of dasatinib on cell migration and invasion of epithelial tumour cells have been well documented in these studies, indicating that dasatinib may not only have anti-proliferative but also anti-invasive and anti-metastatic activity in epithelial tumours.

Our primary aim was to more comprehensively explore the growth-inhibitory effects of this multikinase inhibitor using a large panel of ovarian cancer cell lines, representing all histological subtypes of the disease. To more fully understand the activity, we also studied the effects of dasatinib on cell cycle invasion and apoptosis. Furthermore, we sought to validate previously reported, as well as identify novel response markers, all of which are known to be implicated in Src signalling. Finally, we used multiple drug effect/combination index (CI) isobologram analysis to study the efficacy of chemotherapeutic drugs plus dasatinib combinations tested against dasatinib-sensitive ovarian cancer cells. The current studies were intended to provide a rationale to test dasatinib as a single agent or in combination with chemotherapy in patients with ovarian cancer and to identify molecular markers that may help define subsets of ovarian cancer patients most likely to benefit from treatment with dasatinib.

## Materials and methods

### Cell lines, cell culture, and reagents

The cell lines CAOV3, ES2, OV90, TOV112D, TOV21G, SW626, and OVCAR3 were obtained from the American Type Culture Collection (ATCC, Rockville, MD, USA). The cell lines A2780, OAW28, and OAW42 were obtained from the European Collection of Cell Cultures (Salisbury, UK). The cell lines COLO704, EFO21, and EFO27 were obtained from the German Tissue Repository DSMZ (Braunschweig, Germany). The cell lines MCAS, OVISE, OVKATE, OVMANA, OVSAHA, OVTOKO, and RMG1 were obtained from the Japanese Health Science Research Resources Bank (Osaka, Japan). The cell lines OVCAR5, OV167, OV177, OV207, DOV13, and HEYC2 were a kind gift from Dr V Shridhar (Mayo Clinic, Rochester, MN, USA). The cell line PE06 was a kind gift from Dr SP Langdon (Edinburgh Cancer Research Center, University of Edinburgh, Edinburgh, UK). The cell line HEY was a kind gift from Dr DT Curiel (University of Alabama at Birmingham, Birmingham, AL, USA). The cell lines SKOV3, OV2008, OVCA420, OVCA429, OVCA432, and OVCA433 were a kind gift from Dr B Karlan (Cedars Sinai, Los Angeles, CA, USA). The individuality of each cell line was checked by mitochondrial DNA sequencing. OV167, OV177, and OV207 cells were cultured in MEM medium (Fisher Scientific, Pittsburgh, PA, USA) plus 20% fetal bovine serum (FBS). OVCA420 and OVCA429 cells were cultured in MEM medium (Fisher Scientific) plus 10% FBS. EFO27 and EFO21 cells were cultured in RPMI 1640 medium (ATCC) plus 20% FBS and 1% MEM Non-Essential Amino Acid Solution (ATCC). COLO704, HEY, OV2008, OVISE, OVMANA, OVTOKO, OVKATE, and OVSAHO cells were cultured in RPMI 1640 medium plus 10% FBS. MCAS, DOV13, OVCA432, and OVCA433 cells were cultured in Eagle's minimal essential medium (ATCC) plus 15% FBS. RMG1 cells were cultured in Ham's F12 (ATCC) plus 10% FBS. SW626 cells were cultured in Leibovitz's L-15 medium (ATCC) plus 10% FBS. HEYC2 and PE06 cells were cultured in DMEM/F12 (ATCC) plus 10% FBS. OAW42 and OAW28 cells were cultured in DMEM (ATCC) plus 10%FBS. ES2 and SKOV3 were cultured in McCoy's 5A medium (ATCC) plus 10% FBS. OVCAR5, OVCAR3, and CAOV3 cells were cultured in DMEM medium plus 10% FBS and 4.5 g l^−1^ glucose. A2780 cells were cultured in RPMI 1640 medium plus 10% FBS and 0.25 U ml^−1^ insulin. OV90, TOV21G, and TOV112D cells were cultured in a 1 : 1 mixture of MCDB 105 medium (Cell Applications Inc., San Diego, CA, USA) and Medium 199 (Sigma-Aldrich, St Louis, MO, USA) plus 15% FBS. All cell line cultures were supplemented with 1% penicillin–streptomycin and 1% L-glutamine. Dasatinib was provided by Bristol-Myers Squibb (New York, NY, USA) and prepared as a 10 mmol l^−1^ concentrated stock solution in DMSO.

### Proliferation assays

Cells were plated into 24-well tissue culture plates at a density of 2 × 10^5^ to 5 × 10^5^ and grown in cell-line-specific medium without or with increasing concentrations of dasatinib (ranging between 0.001 and 10 *μ*mol l^−1^). Cells were counted on day 7 using an automated cell viability assay (Vi-CELL XR Cell Viability Analyzer, Beckman Coulter, Fullerton, CA, USA), which is a video imaging system using an automated trypan blue exclusion protocol. Both adherent and floating viable cells were counted for treatment and control wells. Growth inhibition (GI) was calculated as a percentage of untreated controls. The log of the fractional GI was then plotted against the log of the drug concentration and the IC_50_ values were interpolated from the resulting linear regression curve fit (CalcuSyn; Biosoft, Ferguson, MO, USA). Experiments were performed thrice in duplicate for each cell line.

### Cell cycle analysis

Cells were plated in six-well tissue culture plates and treated with vehicle (0.1% DMSO) or 1 *μ*mol l^−1^ dasatinib for 72 h. After collection, the cells were fixed in 70% ethanol, incubated in HCl/Triton X-100, and stained with propidium iodide. Samples were analysed by flow cytometry (Becton Dickinson, San Jose, CA, USA) according to the manufacturer's protocol.

### Annexin V and propidium iodide flow cytometry

Cells were treated with 1 *μ*mol l^−1^ dasatinib for 5 days. Apoptotic subpopulations were detected by labelling phosphatidylserine residues of the cell surface with annexin V-FITC and staining cells with propidium iodide. Detection was carried out on floating and adherent cell fractions by two-colour flow cytometry (Becton Dickinson) according to the manufacturer's protocol.

### Invasion assay

Invasion assays were carried out as previously described (Glynn *et al*, 2004). Briefly, Matrigel (BD Biosciences, San Jose, CA, USA) was diluted to 1 mg ml^−1^ in serum-free medium. A volume of 100 ml of Matrigel was placed into each insert (Falcon) (8.0 mm pore size), which stood in wells of a 24-well plate (Costar, Lowell, MA, USA). The inserts and the plate were incubated for 5 h at 37°C for gelling. Cells were then suspended in serum-free culture media at a concentration of 10^6^ ml^−1^. Subsequently, 100 ml of the cell suspension was added to each insert and 500 ml culture media containing 10% FBS was added to the bottom well. Cells were incubated at 37°C in culture media with or without 1 *μ*mol l^−1^ dasatinib for 48 h. After this time period, the top side of the insert was wiped with a wet swab to remove the cells and the insert was stained with 0.25% crystal violet. Cells entering the Matrigel layer were viewed at × 200 magnification and counted in four random fields per sample. All assays were carried out in triplicate.

### Western blot and immunoprecipitation

Cells were washed in PBS and lysed at 4°C in lysis buffer. Insoluble material was cleared by centrifugation at 10 000 **g** for 10 min. Protein was quantitated using BCA (Pierce, Rockford, IL, USA), resolved by SDS–PAGE, and transferred to nitrocellulose membranes (Invitrogen Life Technologies, Carlsbad, CA, USA). Phosphorylated Src was detected by an anti-pSrc antibody (Tyr416, Calbiochem, San Diego, CA, USA). Phosphorylated FAK was detected using an anti-pFAK antibody (Tyr576/577, Invitrogen Life Technologies). Tyrosine phosphorylation of Yes, Lyn, and Eph2A was analysed as follows. Immunoprecipitations were performed by allowing 250 *μ*g protein lysate to incubate with 3 *μ*g anti-Yes (C-10, Santa Cruz Biotechnology, Santa Cruz, CA, USA), anti-Lyn (H-70, Santa Cruz Biotechnology), and anti-EphA2 antibodies (L-20, Santa Cruz Biotechnology) and protein A/G-agarose (Santa Cruz Biotechnology) at 4°C overnight with gentle agitation. The immunoprecipitates were washed thrice in lysis buffer and then denatured in Laemmli buffer before SDS–PAGE. Immunoblotting was performed using a monoclonal anti-phosphotyrosine antibody (Y20, Becton Dickinson Biosciences, San Jose, CA, USA). Detection was carried out using enhanced chemiluminescence (ECL; Amersham Biosciences UK, Buckinghamshire, England).

### Multiple drug effect analysis

Aliquots of 3 × 10^3^ to 5 × 10^3^ OVCAR3, OVCAR5, RMG1, and SKOV3 cells were plated in 96-well microdilution plates. After cell adherence (24 h), experimental medium containing control medium, dasatinib, carboplatin or paclitaxel, or the combination (dasatinib plus carboplatin or paclitaxel) was added to appropriate wells in duplicate, and serial twofold dilutions were performed to span clinically relevant concentration ranges for the dose-effect analysis for dasatinib and chemotherapeutic agent or drug combination. Multiple drug effect analysis was carried out as previously described (Pegram *et al*, 2004). Combination index values were derived from variables of the median effect plots and statistical tests were applied (unpaired, two-tailed Student's *t*-test) to determine whether the mean CI values at multiple effect levels were significantly different from CI=1. In this analysis, synergy is defined as CI values significantly lower than 1.0, antagonism as CI values significantly higher than 1.0, and additivity as CI values equal to 1.0.

### Gene expression profiling of ovarian cell lines

Agilent microarray analyses were developed for each cell line. Briefly, cells were grown to log phase and then RNA was extracted using the RNeasy Kit (Qiagen, Valencia, CA, USA). The purified RNA was eluted in 30–60 ll DEPC water and the quantity of RNA measured by spectral analysis using the Nanodrop Spectrophotometer. RNA quality was determined by separation of the RNA through capillary electrophoresis using the Agilent 2000 Bioanalyzer (Agilent, Santa Clara, CA, USA). Microarray hybridisations of 34 ovarian cell lines were performed using the Agilent Human 1A V2 array. Characterisation of individual ovarian cancer cell lines by comparison with an ovarian cancer cell line mixed reference pool was conducted on a single slide in which the mixed pool RNA was labelled with cyanine-3 and the individual cell lines with cyanine-5. The mixed reference pool consisted of equal amounts of cRNA from all ovarian cancer cell lines examined. Microarray slides were read using an Agilent Scanner and the Agilent Feature Extraction software version 7.5 was used to calculate gene expression values. The feature-extracted files were imported into the Rosetta Resolver system for gene expression data analysis (version 7.2; Rosetta Biosoftware, Seattle, WA, USA). The intensity ratios between the cell line sample and mixed reference were calculated for each sequence and computed according to the Agilent error model. A particular sequence was considered differentially expressed if the calculated *P*-value was ⩽0.01.

### Statistical methods

The Resolver System ratio-splitting operation was used to generate intensity profiles from the set of ovarian cell line ratio scans and breast cell line ratio scans previously described (Finn *et al*, 2007). The ratio-splitting operation includes error modelling of the channels of each ratio scan, group normalisation, forward transformation of intensities, group detrending, inter-slide error correction, and finally inverse transformation of intensities. After ratio splitting, the ovarian cell lines and breast cell lines were clustered across a set of genes related to dasatinib sensitivity. The two-dimensional cluster analysis was carried out using an agglomerative hierarchical clustering algorithm based on the cosine correlation similarity metric. Before clustering, the intensity values were converted to *Z*-scores using the Resolver System data processing pipeline. The *Z*-score transformation converts the mean for each gene to zero and the standard deviation is set to 1. The cluster data were exported, and associations between the expression levels of biomarkers (*Z*-scores) and GI were analysed using Spearman's *ρ*-correlation, and differences between the subgroups were compared using Student's *t*-test. Cells with ⩾60% GI were classified as highly sensitive, cells with 40–59% GI as moderately sensitive, and cells with <40% GI as resistant. All statistical tests were two-sided.

## Results

### Activity of dasatinib in ovarian cancer cells

**Figure 1:**
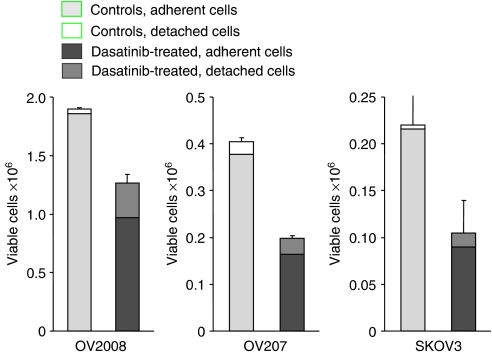
The influence of dasatinib treatment on the detachment of viable cells grown in culture was studied by comparing the fraction of floating cells between dasatinib-treated and control cells. Cells were treated with 0.5 *μ*mol l^−1^ dasatinib. Cells were counted on day 3 by an automated cell viability assay using an automated trypan blue exclusion protocol. Both adherent and floating viable cells were counted for treatment and control wells. Our results indicate that dasatinib can slightly increase the fraction of floating viable cells grown in culture. Therefore, both adherent and floating viable cells were analysed in the cell count experiments used for assessing growth inhibition.

**Figure 2:**
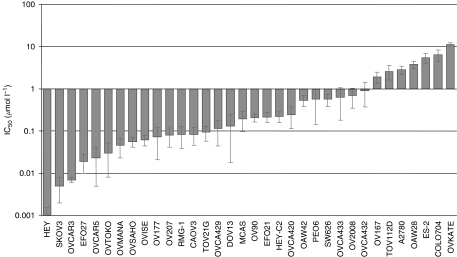
Growth inhibitory effects of dasatinib were studied across a panel of ovarian cancer cell lines. Cells were grown in cell line-specific media without or with increasing doses of dasatinib (ranging between 0.001 and 10 m mol l^−1^). Cells were trypsinised and counted after 7 days of treatment. The percentage of inhibition was calculated compared with untreated controls. The log of the fractional growth inhibition (GI) was plotted against the log of the drug concentration. The dose achieving 50% GI (IC_50_) was interpolated from the resulting linear regression curve fit. Cell lines are ordered from left to right from low to high IC_50_ values. Error bars indicate the s.e. of the mean value. Mean is derived from three replicate experiments.

The effects of dasatinib on human ovarian cancer cells were evaluated using a panel of 34 established human ovarian cancer cell lines. These cells lines were selected to be representative of a range of ovarian cancer subtypes. Of these cell lines, 18 cell lines were obtained from patients with serous papillary ovarian cancer, 8 cell lines from patients with clear cell ovarian cancer, and 4 cell lines from patients with undifferentiated ovarian cancer. The remaining four cell lines were obtained from patients with endometrioid or mucinous ovarian cancer. Earlier reports indicate that dasatinib may inhibit cell-surface adhesion of epithelial tumour cells grown in culture (Nam *et al*, 2005; Serrels *et al*, 2006). To study the influence of dasatinib treatment on the detachment of viable cells grown in culture, we compared the fraction of floating cells between dasatinib-treated and control cells. Our results indicate that dasatinib can slightly increase the fraction of floating viable cells grown in culture (Figure 1). Therefore, both adherent and floating viable cells were analysed in the cell count experiments used for assessing GI. The effective dose range (IC_10_–IC_80_) was identified using a wide range of dasatinib concentrations (0.001–10 *μ*mol l^−1^). Dasatinib inhibited the proliferation of all ovarian cancer cell lines investigated in a concentration-dependent manner; however, the IC_50_ values varied significantly between individual cell lines with up to a 3 log-fold difference in the IC_50_ values and ranged between 0.001 *μ*mol l^−1^ in HEY ovarian cancer cells and 11.3 *μ*mol l^−1^ in OVKATE ovarian cancer cells (Table 1 and Figure 2). When comparing the sensitivity with dasatinib between histological subtypes, none of the serous papillary, clear cells, or the endometrioid, mucinous, or undifferentiated ovarian cancer cells were preferentially sensitive to dasatinib (data not shown). We have previously reported on the growth inhibitory effect of dasatinib in a large representative panel of 39 breast cancer cell lines (Finn *et al*, 2007). When treated with dasatinib *in vitro*, 8 (21%) of them were highly sensitive (⩾60% GI), 10 of them were moderately sensitive (40–59% GI), and 21 were resistant (<40% GI) to dasatinib. The results of this study indicate that 24 out of the 34 (71%) ovarian cancer cell lines were highly sensitive (⩾60% GI), 6 of them were moderately sensitive (40–59% GI), and only 4 were resistant to dasatinib (<40% GI). Importantly, we used the identical cell proliferation assays for assessing GI in both the breast and the ovarian cancer cell line panels.

### Predictors of dasatinib response *in vitro*

**Figure 3:**
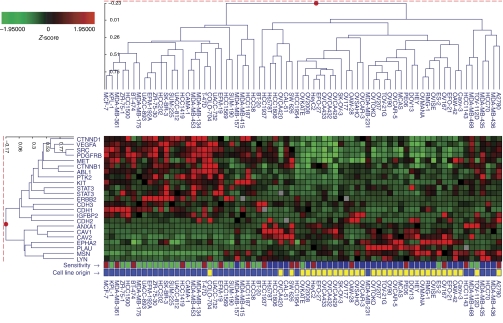
Microarray hybridisations of 34 ovarian cell lines were performed using the Agilent Human 1A V2 array. Characterisation of individual ovarian cancer cell lines by comparison with an ovarian cancer cell line mixed reference pool was conducted on a single slide in which the mixed pool RNA was labelled with cyanine-3 and the individual cell lines with cyanine-5. The mixed reference pool consisted of equal amounts of cRNA from all ovarian cancer cell lines examined. The Resolver System was used to generate intensity profiles from the set of ovarian cell line ratio scans and breast cell line ratio scans previously described (Finn *et al*, 2007). The red and green matrices represent the normalised expression patterns for each gene in Table 2 across the 34 ovarian and 39 breast cancer cell lines. Brightest red indicates the highest relative expression and brightest green indicates the lowest relative expression. The bottom 2 rows are matrices that represent the *in vitro* sensitivity to dasatinib (blue=highly sensitive, ⩾60% growth inhibition (GI); red=moderately sensitive, 40–59% GI; and green=resistant, <40% GI), and the cell line origin (yellow=ovarian cancer cell line; blue=breast cancer cell line).

A key aspect of therapies with targeted agents is the accurate selection of patients most likely to benefit from therapy. Earlier reports have shown that high expression of annexin-1, caveolin-1, caveolin-2, moesin, and uPA, as well as low expression of IGFBP2, was associated with the *in vitro* response to dasatinib in breast, lung, and prostate cancer cells (Finn *et al*, 2007; Huang *et al*, 2007; Wang *et al*, 2007). Here, we explored the association between the various biomarkers implicated in Src signalling pathways and the *in vitro* response to dasatinib in 73 human cancer cell lines, including the current 34 ovarian cancer cell lines and 39 breast cancer cell lines that have been published earlier (Finn *et al*, 2007). Restricting such a correlative analysis between the *in vitro* response and differential gene expression to only ovarian cancer cell lines would be of limited value as most ovarian cancer cell lines were highly sensitive and only four ovarian cancer cell lines showed resistance (defined as <40% GI) and only seven ovarian cancer cell lines showed an IC_50_ >1 *μ*M. Importantly, *in vitro* sensitivity towards dasatinib was assessed using the same methodology in the ovarian and breast cancer cell line panels, and cell lines were uniformly classified as highly sensitive (⩾60% GI), moderately sensitive (40–59% GI), and resistant to dasatinib (<40% GI). Of the known dasatinib targets, high expression of Yes, Lyn, and EphA2 but not of Src, FAK, Kit, or PDGFR-*β* was associated with *in vitro* sensitivity to dasatinib (Figure 3, Table 2). Furthermore, our findings corroborate earlier data, in that cell lines with high expression of annexin-1, caveolin-1, caveolin-2, moesin, and uPA but low expression of IGFBP2 were the most sensitive to dasatinib (Figure 3, Table 2). We also studied additional markers such as E-cadherin, P-cadherin, and N-cadherin that have an important role in cell adhesion, as well as *β*-catenin and *δ*-catenin/p120, both found in complexes with cadherin molecules. Our results indicate that cell lines with high expression of E-cadherin or P-cadherin, as well as *δ*-catenin/p120, were less sensitive to dasatinib when compared with those with lower expression of the respective markers (Figure 3, Table 2). We also studied the expression of receptor tyrosine kinases such as HER2 and MET or components of the VEGF/STAT3 signalling pathway, which can all signal through Src (Mukhopadhyay *et al*, 1995; Rahimi *et al*, 1998; Laird *et al*, 2003; Vadlamudi *et al*, 2003). Surprisingly, high expression of HER2, MET, or VEGF and STAT3 was not associated with *in vitro* sensitivity to dasatinib; in fact, high expression of HER2, VEGF, and STAT3 was rather correlated with *in vitro* resistance to dasatinib.

### Effects of dasatinib on ovarian cancer cell cycling, survival, and invasion

**Figure 4:**
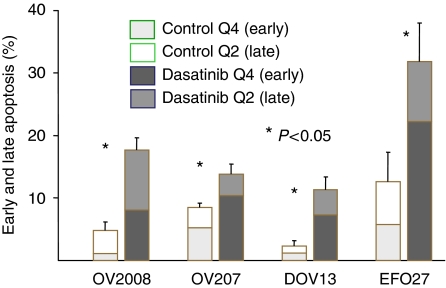
Detection of apoptotic subpopulations was achieved by labelling phosphatidylserine residues of the cell surface with annexin V-FITC and staining cells with propidium iodide. Cells were treated with 1 *μ*mol l^−1^ dasatinib for 5 days. This staining allows differentiation between early (Q4) and late (Q2) apoptotic subpopulations.

**Figure 5:**
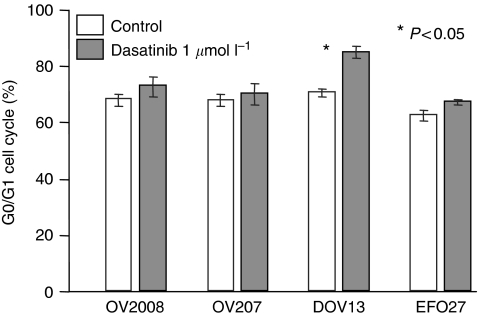
Cell cycle analysis of cells treated with dasatinib. Cells were treated with vehicle (0.1% DMSO) or 1 *μ*mol l^−1^ dasatinib for 72 h. Cells were analysed by flow cytometry after propidium iodide staining.

**Figure 6:**
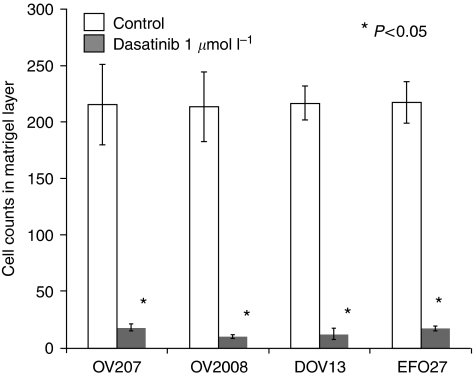
Invasion was assessed using Matrigel invasion assays as described in Materials and methods. Cells were incubated at 37°C in culture media with or without 1 *μ*mol l^−1^ dasatinib for 48 h. Cells invading the Matrigel layer were viewed at × 200 magnification and counted in four random fields per sample. All assays were performed in triplicate.

Earlier reports have suggested that dasatinib may have anti-proliferative activity by inducing cell cycle arrest and apoptosis in lung or head and neck cancer cells (Johnson *et al*, 2005; Song *et al*, 2006), malignant pleura mesothelioma (Tsao *et al*, 2007), or melanoma cells (Eustace *et al*, 2008). Here, we confirm and extend these findings to ovarian cancer cells. At a concentration of 1 *μ*mol l^−1^, dasatinib treatment significantly increased the fraction of early and late apoptotic cells in the human ovarian cancer cell lines examined (Figure 4). In contrast, however, treatment with 1 *μ*mol l^−1^ dasatinib over 72 h only lead to a modest increase in the fraction of cells in the G0/G1 cell cycle phase in only one out of the four examined human ovarian cancer cell lines when compared with the untreated controls (Figure 5). At a concentration of 1 *μ*mol l^−1^, dasatinib treatment also significantly inhibited the ability of ovarian cancer cells to invade matrigel when compared with untreated controls in all of the four examined cell lines (Figure 6).

### Effects of dasatinib on Src, Yes, Lyn, FAK, and Eph2A phosphorylation

**Figure 7:**
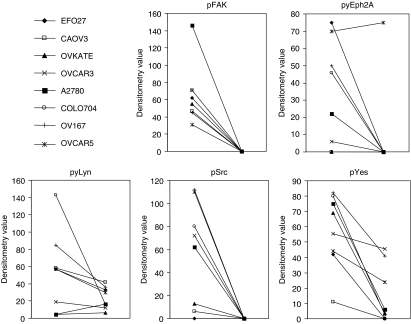
Effect of dasatinib on the phosphorylation state of Src, Lyn, Yes, EphA2, and FAK using immunoblotting techniques. Exposure of EFO27, CAOV3, OVKATE, OVCAR3, A2780, COLO704, OV167, and OVCAR5 cells to 1 *μ*mol l^−1^ dasatinib for 1 h resulted in a pronounced reduction of pSrc, pYes, pLyn, pEphA2, and pFAK.

We assessed the effect of dasatinib on the phosphorylation state of Src, Lyn, Yes, EphA2, and FAK using immunoblotting techniques. Exposure of EFO27, CAOV3, OVKATE, OVCAR3, A2780, COLO704, OV167, and OVCAR5 cells to 1 *μ*mol l^−1^ dasatinib for 1 h resulted in a pronounced reduction of pSrc, pYes, pLyn, pEphA2, and pFAK (Figure 7).

### Combination of dasatinib and chemotherapeutic agents

**Figure 8:**
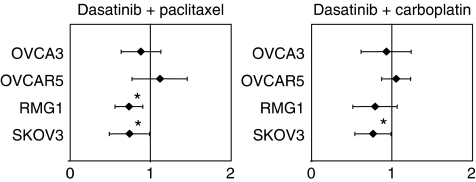
Mean CI values for chemotherapeutic drug–dasatinib combinations in four different human ovarian cancer cell lines. Error bars indicate the 95% confidence interval of the mean value. Mean is derived from three replicates spanning clinically relevant concentration ranges sufficient to inhibit growth of control cells by 20–90%. Combination index values were derived from parameters of the median effect plots, and statistical tests were used to determine whether the CI values at multiple effect levels (IC_20_–IC_90_) were statistically significantly different from CI values equal to 1. Values that are statistically significantly <1 indicate synergistic interactions. Values that are statistically significantly >1 indicate antagonistic interactions. Values equal to (or not statistically significantly different from) 1 indicate additive interactions.

We next analysed the combination of dasatinib and chemotherapeutic agents used for the treatment of ovarian cancer. Multiple drug effect analysis was performed using four dasatinib-sensitive ovarian cancer cell lines to determine the nature of the interaction between dasatinib and carboplatin or paclitaxel (synergy, addition, or antagonism). The dasatinib concentrations used for these experiments ranged between 0.031 and 0.5 *μ*mol l^−1^ for OVCAR5, 0.050 and 0.8 *μ*mol l^−1^ for SKOV3 cells, 0.008 and 0.125 *μ*mol l^−1^ for RMG1, and 0.016 and 0.250 *μ*mol l^−1^ for CAOV3 cells and were below the reported peak plasma concentrations achievable in humans (Wang *et al*, 2008). The drug concentrations of carboplatin and paclitaxel used for these experiments were similarly below the reported peak plasma concentrations that have been previously published (Pegram *et al*, 2004). Synergistic and additive interactions were observed for dasatinib plus carboplatin (mean CI values ranged from 0.73 (95% CI: 0.56–0.90, *P*=0.002) in RMG1 cells to 1.11 (95% CI: 0.77–1.46, *P*=0.553) in OVCAR5 cells) and for dasatinib plus paclitaxel (0.76 (95% CI: 0.54–0.99, *P*=0.016) in SKOV3 cells to 1.05 (95% CI: 0.87–1.23, *P*=0.524) in OVCAR5 cells) (Figure 8).

## Discussion

Although the Src kinase has been linked with the development and progression of cancer for many years, there has only recently been a renewed interest in this onco-protein as a molecular target for cancer therapy. Src family kinases modulate signal transduction through multiple oncogenic pathways, including EGFR, HER2, PDGFR, FGFR, and VEGFR (Yeatman, 2004). In turn, a wide range of Src substrates have been identified that regulate cell growth, adhesion, invasion, and motility (Thomas and Brugge, 1997; Yeatman, 2004). Thus, it is anticipated that blocking Src kinase would be predicted to have a broad therapeutic benefit in patients with Src-dependent cancers. Dasatinib is currently being tested or entering clinical trials in various epithelial malignancies (http://ClinicalTrials.gov). However, despite the ubiquitous expression of SFKs, they may have varying roles in different epithelial cancers and, moreover, may be more important in specific disease subgroups compared with others. Here, we are able to show that approximately two-thirds of the ovarian cancer cell lines examined showed high *in vitro* sensitivity to dasatinib, which is in stark contrast to the fact that similar sensitivity was only seen in one-fifth of the breast cancer cell lines examined. Nevertheless, we still found a wide range of *in vitro* response to dasatinib with up to a threefold log difference in IC_50_ values between the most sensitive and the most resistant cell lines. We therefore sought to validate biomarkers that predict *in vitro* response to dasatinib. In doing so, we focused primarily on biomarkers that have been reported earlier or that are known to be implicated in Src signalling. We intended to identify a limited number of individual response markers that may be more useful for independent validation in future clinical studies. Our results confirm data from earlier studies that were obtained in breast, lung, and prostate cancer cell lines, in that high expression levels of caveolin-1, caveolin-2, annexin-1, moesin, Eph2A, and uPA were associated with *in vitro* sensitivity to dasatinib (Finn *et al*, 2007; Huang *et al*, 2007; Wang *et al*, 2007). Caveolins are membrane proteins involved in receptor-independent endocytosis and can be upregulated during epithelial-to-mesenchymal transition (Bailey and Liu, 2008). Moesin, a member of the ezrin/radixin/moesin family of actin-binding proteins, cross-links the actin cytoskeleton to the plasma membrane (Rosenblatt, 2008) and recent data suggest that cell movement through rapid actin cytoskeleton remodelling is mediated by moesin activation (Fu *et al*, 2008). Thus, high expression of these two markers that are involved in cell adhesion and migration may characterise a cell phenotype that is particularly susceptible to Src inhibition. Moreover, of the known dasatinib targets, increased expression of Eph2A, Yes, and Lyn was associated with *in vitro* sensitivity to dasatinib.

Normal epithelial architecture and function are maintained by integrins and cadherins, transmembrane receptors that mediate cell–ECM and cell–cell interactions, respectively. Importantly, signalling centres organised by these receptors modulate anchorage dependence and contact-dependent inhibition of cell growth, which are essential cellular functions often disrupted in cancer. E-cadherin is the main cell–cell adhesion molecule in epithelial tissues and is regarded as a master organiser of the epithelial phenotype. E-cadherin is regulated in part by cytoplasmic binding partners called catenins (*β*-catenin, and p120-catenin). *β*-Catenins are primarily associated with physical and/or functional linkage to the actin infrastructure (Yamada *et al*, 2005), whereas p120-catenin appears to modulate cadherin stability at the cell surface (Ireton *et al*, 2002; Reynolds and Roczniak-Ferguson, 2004). Thus, high expression of E-cadherin and p120-catenin, which are hallmarks of an epithelial cell phenotype, may characterise tumour cells that are less susceptible to Src inhibition.

Our study also confirms that low expression of IGFBP2 may be associated with *in vitro* sensitivity to dasatinib (Huang *et al*, 2007). Conversely, high expression of IGFBP2 may thus characterise a cell phenotype less responsive to the inhibition of Src- or FAK-driven transformation. Recent data support this hypothesis, in that IGFBP2 was shown to independently promote cell mobility through its interaction with integrins (Schütt *et al*, 2004; Wang *et al*, 2006). Moreover, IGFBP2 has been described to be a candidate biomarker for PTEN status and PI3K/Akt pathway activation (Mehrian-Shai *et al*, 2007).

Dasatinib was approved for the treatment of imatinib-resistant/intolerant patients with CML or Philadelphia chromosome-positive acute lymphoblastic leukaemia at the dosage of 70 mg twice daily. However, toxicity issues have hampered the clinical development of this compound in epithelial malignancies. Nevertheless, a phase III dose-optimisation study has recently been carried out to compare the aforementioned regimen with others, including dasatinib 100 mg once daily, in patients with CML (Tyler, 2009). The results of this study showed that there was no significant difference in efficacy between these two regimens. The safety profile was improved in the 100-mg once-daily dasatinib arm with significantly reduced frequencies of grade 3–4 thrombocytopenia and all-grade pleural effusions. The number of patients who had to discontinue, reduce, or interrupt their dosage was also less among patients taking dasatinib 100 mg once daily (Tyler, 2009).

To determine how best to use dasatinib either as a single agent or in combination with chemotherapy, we conducted a series of *in vitro* studies to evaluate its inhibitory effects in combination with carboplatin or paclitaxel. These preclinical studies have shown significantly enhanced activity when chemotherapy is combined with dasatinib in ovarian cancer cells. Taken together, our findings support further clinical evaluation of dasatinib as a single agent or in combination with chemotherapy in patients with ovarian cancer. Moreover, the assessment of functionally implicated response predictors in these clinical trials may help to identify the patient subgroup most likely to benefit from treatment with dasatinib.

## Figures and Tables

**Table 1:** Dasatinib concentrations that achieve IC_50_ and percent growth inhibition compared with untreated controls at a fixed concentration of 1 *μ*mol l^−1^ dasatinib

**Cell line**	**IC_50_ (*μ*mol)**	**s.e.**	**Growth inhibition in % at 1 *μ*mol**	**s.e.**	**Histology**
HEY	0.001	0.001	95	1.1	Serous
SKOV3	0.005	0.004	82	1.3	Serous
OVCAR3	0.007	0.001	75	8.0	Serous
EFO27	0.019	0.009	84	4.1	Mucinous
OVCAR5	0.023	0.018	71	4.9	Undifferentiated
OVTOKO	0.030	0.022	81	5.8	Clear cell
OVMANA	0.046	0.023	66	7.7	Clear cell
OVSAHO	0.056	0.014	69	10.1	Serous
OVISE	0.062	0.017	73	3.8	Clear cell
OV177	0.072	0.051	67	6.7	Serous papillary
OV207	0.080	0.038	69	1.9	Clear cell
RMG-1	0.082	0.043	71	4.6	Clear cell
CAOV3	0.084	0.038	68	4.8	Serous
TOV21G	0.094	0.036	88	0.4	Clear cell
OVCA429	0.113	0.068	68	7.0	Clear cell
DOV13	0.130	0.112	80	6.7	Serous
MCAS	0.196	0.097	67	5.3	Mucinious
OV90	0.209	0.043	78	3.1	Serous
EFO21	0.216	0.057	64	5.7	Serous
HEY-C2	0.221	0.062	55	3.5	Serous
OVCA420	0.243	0.127	78	4.6	Serous
OAW42	0.538	0.149	64	5.3	Serous
PEO6	0.562	0.416	48	5.4	Serous
SW626	0.565	0.179	58	0.4	Undifferentiated
OVCA433	0.624	0.440	70	7.0	Serous
OV2008	0.688	0.334	63	4.5	Endometrioid
OVCA432	0.906	0.530	66	7.7	Serous
OV167	1.905	0.587	52	10.3	Serous
TOV112D	2.565	0.953	34	9.1	Endometrioid
A2780	2.829	0.638	44	5.5	Undifferentiated
OAW28	3.760	0.742	43	2.1	Serous
ES-2	5.519	1.436	28	7.4	Clear cell
COLO704	6.428	1.996	29	5.6	Undifferentiated
OVKATE	11.229	1.229	25	4.7	Serous

**Table 2:** Association between *in vitro* growth inhibition after dasatinib treatment (at a concentration of 1 *μ*mol l^−1^) and the relative expression of biomarkers implicated in Src signaling pathways in 34 ovarian and 39 breast cancer cell lines (Finn *et al*, 2007)

**Name**	**Symbol**	**Correlation coefficient** a	***P*-value**
Urokinase plasminogen activator	UPA	0.63	<0.001
Moesin	MSN	0.60	<0.001
Caveolin-2	CAV2	0.57	<0.001
Caveolin-1	CAV1	0.54	<0.001
Ephrin A2	Eph2A	0.53	<0.001
Annexin-1	ANAX1	0.53	<0.001
Yamaguchi sarcoma viral related oncogene homolog	LYN	0.41	0.016
Yamaguchi sarcoma viral oncogene homolog 1	YES	0.36	0.002
Hepatocyte growth factor receptor	MET	0.19	0.111
N-cadherin	CDH2	0.19	0.105
Beta-catenin	CTNNB1	−0.10	0.390
v-src sarcoma viral oncogene homolog	SRC	−0.23	0.046
Signal transducer and activator of transcription-3	STAT3	−0.26	0.029
c-abl	ABL	−0.27	0.021
P-cadherin	CDH3	−0.34	0.003
Focal Adhesion Kinase	PTK2	−0.35	0.003
E-cadherin	CDH1	−0.38	0.001
c-kit	KIT	−0.41	<0.001
Human epidermal growth factor receptor-2	HER2	−0.43	<0.001
Vascular endothelial growth factor	VEGFA	−0.43	<0.001
Insulin-like growth factor binding protein-2	IGFBP2	−0.47	<0.001
Platelet derived growth factor receptor-*β*	PDGFRB	−0.55	<0.001
Delta-catenin/p120	CTNND1	−0.55	<0.001

Cell lines were classified as highly sensitive (⩾60% growth inhibition, moderately sensitive (40–59% growth inhibition), and resistant (<40% growth inhibition).

a Spearman's *ρ*-correlation.
